# Auer Rods Are Not Seen in Non-Neoplastic Cells

**DOI:** 10.4274/tjh.2016.0179

**Published:** 2016-12-01

**Authors:** İrfan Yavaşoğlu, Zahit Bolaman

**Affiliations:** 1 Adnan Menderes University Faculty of Medicine, Division of Hematology, Aydın, Turkey

**Keywords:** Auer rods, Non-neoplastic cells

## To the Editor,

The article entitled “Auer Rod in a Neutrophil in a Nonmalignant Condition”, written by Chandra et al. [1] and published in a recent issue of your journal, was quite interesting. Here we would like to emphasize some relevant points.

This article demonstrates why peripheral smears, bone marrow examination, and genetic tests are mandatory. Acute myeloid leukemia must be excluded. Electron microscopic analyses would be helpful. The title is overly assertive. It may be called an Auer rod-like image.

It is not known why Auer rods are not seen in non-neoplastic cells. However, there have been some hypotheses on the genesis of Auer rods, including infectious microorganisms, abnormal nucleoplasm segregation, pathologic forms of azurophilic granules, and cytoplasmic pH alteration. Unsuccessful results in Auer body inoculation experiments led to the elimination of the infectious microorganism theory. Although the conditions for pH alteration are not known, Ackerman [2] suggested that cytoplasmic pH alteration occurred in specific leukemia cells, which allowed the granules to unite into crystal-like rods [3].

Additionally, a titration rate of 1/200 or higher in O antigen should be considered positive for acute infection diagnosis. Salmonella Typhi isolation in culture is the gold standard for diagnosis [4].

Conflict of Interest: The authors of this paper have no conflicts of interest, including specific financial interests, relationships, and/or affiliations relevant to the subject matter or materials included.
