# Supplementary material for: Auer Rods Are Not Seen in Non-Neoplastic Cells
Source: Turk J Haematol. 2016 Dec 1;33(4):368–9. doi: 10.4274/tjh.2016.0179 (PMC5204204; doi:10.4274/tjh.2016.0179)
Supplement: Related texts

# Reply: Authors' Response

**Authors:** Harish Chandra, Smita Chandra, Vibha Gupta, Divyaa Mahajan

## Dear Sir,

The authors are thankful for considering their manuscript entitled “Auer Rod in a Neutrophil in a Nonmalignant Condition” interesting enough for critical analysis. However, the authors would like to clarify few points:

1. The authors have clearly stated in the manuscript the presence of Auer rod-like inclusions on peripheral examination.

2. In view of the presence of Auer rods bone marrow examination was done and which showed only unremarkable features of normoblastic maturation. There was presence of no leukemia cells. This clearly excluded the possibility of malignant condition and nonmalignant diagnosis was considered. Moreover the patient also responded well to an antibiotic course after diagnosis of typhoid.

3. Genetic studies were however not done as firstly it was not considered necessary in view of absolutely normal bone marrow and secondly also due to financial constraints.

4. The Salmonella Typhi O antigen titre of 1: 160 dilution was considered positive and patient responded very well to course of antibiotics. Her clinical follow up was unremarkable and thus chance of any leukemic process was completely eliminated.

5. The authors agree with the various hypotheses that have been enlisted by Yavaşoğlu and Bolaman for genesis of Auer rod in infectious conditions. In the background of these theories and findings, the presence of clear rod-like structure due to condensation of azurophilic granules in neutrophil in typhoid infection led to the consideration of Auer rod in nonmalignant condition.

Thanking you,
